# Exploring Individual Differences in Recognizing Idiomatic Expressions in Context

**DOI:** 10.5334/joc.183

**Published:** 2021-08-12

**Authors:** Mesian Tilmatine, Ferdy Hubers, Florian Hintz

**Affiliations:** 1 Free University Berlin, Berlin, DE; 2 Centre for Language Studies, Radboud University, Nijmegen, NL; 3 Max Planck Institute for Psycholinguistics, Nijmegen, NL

**Keywords:** idiomatic expressions, individual differences, self-paced reading

## Abstract

Written language comprehension requires readers to integrate incoming information with stored mental knowledge to construct meaning. Literally plausible idiomatic expressions can activate both figurative and literal interpretations, which convey different meanings. Previous research has shown that contexts biasing the figurative or literal interpretation of an idiom can facilitate its processing. Moreover, there is evidence that processing of idiomatic expressions is subject to individual differences in linguistic knowledge and cognitive-linguistic skills. It is therefore conceivable that individuals vary in the extent to which they experience context-induced facilitation in processing idiomatic expressions. To explore the interplay between reader-related variables and contextual facilitation, we conducted a self-paced reading experiment. We recruited participants who had recently completed a battery of 33 behavioural tests measuring individual differences in linguistic knowledge, general cognitive skills and linguistic processing skills. In the present experiment, a subset of these participants read idiomatic expressions that were either presented in isolation or preceded by a figuratively or literally biasing context. We conducted analyses on the reading times of idiom-final nouns and the word thereafter (spill-over region) across the three conditions, including participants’ scores from the individual differences battery. Our results showed no main effect of the preceding context, but substantial variation between readers and variation in contextual facilitation. We encourage interested researchers to exploit the present dataset for follow-up studies on individual differences in idiom processing.

## Introduction

To understand sentences and discourse properly, readers must know facts about the world and the plausibility of a described situation. In some cases, retrieving and combining the meaning of individual words is not sufficient to activate the meaning intended by a sentence or discourse. That is, there are cases where fixed sequences of words, also known as instances of formulaic language, carry a meaning that does not emerge from its constituent words and that differ from the literal interpretation of the word sequence (Abel, 2003).

A prominent type of fixed word sequences are idiomatic expressions (Wray & Perkins, 2000). Previous research on idioms has shown that they are often processed faster than regular expressions, because they are well-known, pre-established sequences of words that can be predicted (Tabossi, Fanari & Wolf, 2009). An extensive debate in the field has concerned the mental representation and processing of idioms. According to the ‘lexical representation hypothesis’, the meaning of idiomatic expressions is represented as a single unit rather than being composed ‘on the fly’ (Bobrow & Bell, 1973; Swinney & Cutler, 1979; Gibbs, 1980). In contrast, compositional approaches assume that each constituent word contributes to the meaning of an idiomatic expression (Gibbs & O’Brien, 1990; Cacciari & Glucksberg, 1991, McGlone, Glucksberg, & Cacciari, 1994). Finally, hybrid models (e.g., Titone and Connine, 1999), representing a mixture of lexical representation and compositional accounts, have received a lot of empirical support and are nowadays widely accepted. Such models assume that there are external forces (e.g., idiom frequency, discourse context, language user characteristics) that act upon the precise nature of an idiom’s meaning activation. A good test case for examining the predictions of idiom processing and storage accounts are ‘literally plausible expressions’.

Literally plausible idiomatic expressions, such as ‘to play with fire’, are cases where both the literal (playing with fire) and the figurative (taking a risk) meaning of the expression are frequently used. It has been shown that in order to select the intended meaning from the two alternatives, readers make use of context, which may bias either the figurative or the literal interpretation (Cacciari & Tabossi, 1988; Holsinger, 2013).

Beck and Weber (2020) conducted a self-paced reading study to investigate the effects of context on the processing of idiomatic expressions. Their participants read idioms embedded in sentences that varied in how literally plausible the idiom was and in whether the preceding context was figuratively or literally biasing. The idioms were followed by a resolution phrase that indicated whether the intended reading was figurative or literal. An example item with consistent resolution phrases is given in (1): the biasing context is in italics, the idiom is bold font, and the resolution phrase is underlined. Their results showed that both types of context facilitated processing when the resolution phrase was consistent with the intended figurative or literal interpretation as compared to when it was inconsistent. However, contexts biasing a literal interpretation facilitated processing only in idioms that had a high potential for a literal interpretation.

**Table:** 

(1)	*a.*	*The fearless climber, who was on a climb alone in the mountains*, was ready to **play with fire** with any risk if necessary later on.
	*b.*	*The young camper, who was already bored without any of his friends*, was ready to **play with fire** from the grill if necessary later on.

Individuals vary substantially in their ability to use language (Dąbrowska, 2018; Kidd et al., 2018). A recent report demonstrated that fluid and crystallized intelligence predicted the comprehension of metaphors (Stamenković, Ichien, & Holyoak, 2019), a form of figurative language. It is therefore conceivable that language users also differ in their ability to process idiomatic expressions.

In a first step towards exploring skills that underlie individual differences in idiom processing, Cacciari, Corrardini, and Ferlazzo (2018) conducted a cross-modal priming experiment. Their participants heard idioms embedded in a sentence context that biased the figurative interpretation of the idiom. Following auditory presentation, a written target word that was semantically related to the idiom appeared on the screen. Participants performed a lexical decision task on the written word. The underlying assumption was that participants who recognize an idiom quickly respond faster to the semantically related target word due to spreading activation. Cacciari and colleagues tested whether variability in lexical decision times could be explained by measures of participants’ non-verbal processing speed, inhibitory control, working memory, cognitive flexibility, crystallized and fluid intelligence, and personality traits. In terms of linguistic and general cognitive skills, their analyses showed positive effects of working memory, inhibitory control and crystallized verbal intelligence on lexical decision times (i.e., reflected in shorter RTs).

In sum, previous research has shown that processing is facilitated when idioms are embedded in contexts biasing either a figurative or a literal interpretation—in the latter case, only if the idiom is literally plausible. Moreover, there is evidence from a cross-modal priming paradigm for individual differences in idiom processing, which have been related to differences in linguistic and general cognitive skills.

One open question resulting from this body of research concerns the extent to which contextual facilitation in idiom processing is subject to individual differences. That is, given the results by Beck and Weber (2020) demonstrating facilitatory effects of context on self-paced reading times as well as the individual differences data by Cacciari et al. (2018), it is likely that there is considerable individual variation in how figuratively and literally biasing contexts affect readers’ processing of idiomatic expressions. The present study addressed this question. We ran a self-paced reading experiment via the internet using Dutch idioms selected from the normative idiom database by Hubers et al. (2018, 2019). Next to their figurative meaning, all idioms had a high potential for being interpreted literally. The idioms were embedded in short sentences. Our analyses focused on the reading times of the idiom-final noun, the most meaning-bearing element in the fixed expression (see Rommers et al., 2013; e.g., the word ‘fire’ in (1)). To allow for analyses of spill-over effects (Mitchell, 1994), we added a neutral adverb to follow the idiom-final noun, which marked the end of the sentence. Participants read the sentences word by word in a non-cumulative, stationary window, self-paced fashion. Importantly, each participant read each idiom in all of the three conditions: without a preceding context (to assess the baseline reading time), or preceded by either a figuratively or literally biasing context. This within-participants manipulation enabled us to determine for each participant to what extent context affected their reading of the idiom. Moreover, since the experiment was conducted via the internet, we expected large variation between participants pertaining to the speed of their internet connection and the quality of their hardware (e.g., keyboard polling rate). We reasoned that a within-participants design would mitigate these sources of noise as hardware-related noise should be constant across conditions. On the other hand, repeating the same idiom twice within a participant – albeit that the order of conditions was counterbalanced across lists – might affect their processing of the idiom. We therefore offer two analyses—one based on the first encounter an idiom (in one of the three conditions), and one based on the full dataset (including the two item reputations, Appendix C). In general, we want to stress that the primary goal of this data report is to provide a brief motivation and sample analysis for the present data. Interested researchers may further exploit the dataset for targeted and/or exploratory analyses.

Our participants were native speakers of Dutch, who had recently taken part in a large-scale individual-differences study where they completed 33 tests measuring linguistic and general cognitive skills (Hintz et al., 2020). Hintz et al. (2020) used a latent-variable approach with multiple tests tapping into the same cognitive construct. For the present analyses, we selected 19 of the 33 tests^1^ that appeared relevant in the context of present study (cf. Cacciari et al., 2018). Specifically, the selected tests tapped into five cognitive constructs: (1) Linguistic experience, (2) Non-verbal processing speed, (3) Visual working memory, (3) Non-verbal intelligence, (4) Word reading skills, and (5) Predictive sentence comprehension skills. We used principal component analysis to derive one score for each of the 112 participants and each construct to be used in the analyses predicting idiom-final word and spill-over reading times.

We predicted that compared to the condition where idioms were read in isolation, figuratively and literally biasing contexts should lead to faster reading of idiom-final and spill-over words (Beck & Weber, 2020). The crucial question was if and how individual differences in linguistic and general cognitive skills affect idiom processing in context. While readers with higher levels of non-verbal processing speed, non-verbal intelligence, and word reading skills may have a general processing advantage (affecting reading times in all three conditions) over readers with lower scores on these tests, the influence of visual working memory may be restricted to both context conditions. That is, readers with larger visual working memory capacity may remember and use preceding contexts more efficiently than readers with lower capacities. Similarly, readers with extensive linguistic experience (e.g., vocabulary size, reading frequency) may have encountered idioms in a variety of different contexts and may thus be faster at processing idioms in contexts than readers with less linguistic experience. Finally, readers with better prediction skills during sentence comprehension may be able to exploit the preceding contexts more efficiently for generating predictions about upcoming idiom-final words than readers with worse prediction skills.

## Method

### Principal component analyses of linguistic and general cognitive skills

**Figure 1:**
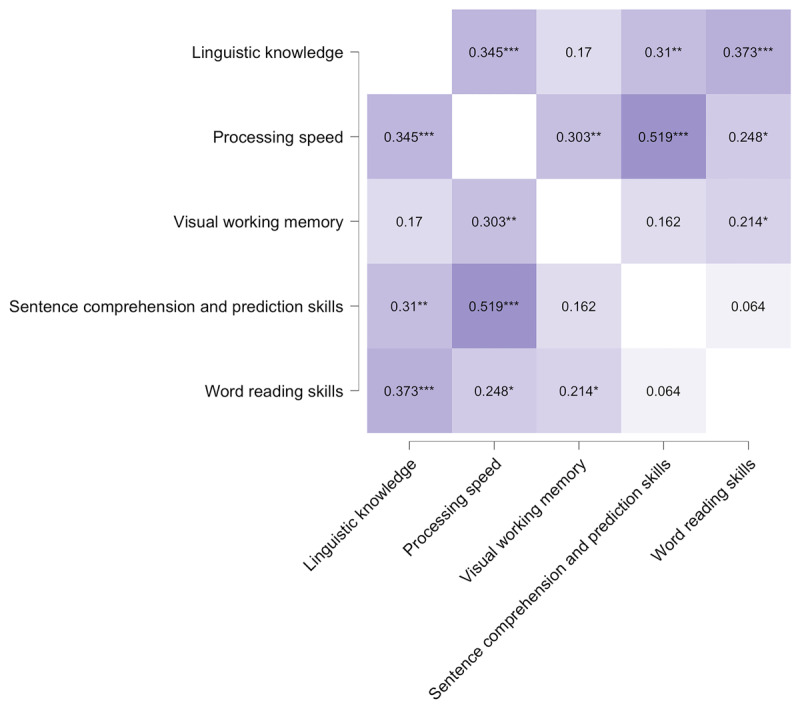
Correlations between individual differences predictors.

Before running the present study, we conducted a principal component analysis (using SPSS, version 27) on the test scores provided by Hintz et al. (2020). That is, for each of the five constructs (linguistic knowledge, processing speed, visual working memory, sentence comprehension and prediction skills, word reading skills), we tested how strongly the tests assumed to measure a given construct loaded on its factor and how much variance was explained (***Table 1***; see Appendix A for descriptive statistics and reliability measures of each included test and ***Figure 1*** for correlations between the predictor variables). The scores from Raven’s Advanced Progressive Matrices test served as the measure of non-verbal intelligence. We selected ‘oblimin rotation’ and extracted regression-based factor scores.

**Table 1:** Linguistic and general cognitive constructs: Factor loadings and variance explained.


CONSTRUCT	N	EXPL. VARIANCE	INCLUDED TESTS	LOADING

Linguistic knowledge	112	58%	Peabody Picture Vocabulary Test	0.84

			Spelling	0.75

			Dutch Author Recognition Test	0.82

			Idiom recognition	0.54

			Prescriptive grammar	0.83

Processing speed	107	53%	Auditory simple reaction time	0.71

			Auditory choice reaction time	0.83

			Letter comparison	0.48

			Visual simple reaction time	0.74

			Visual choice reaction time	0.81

Visual working memory	106	30%	Corsi block clicking forward	0.82

			Corsi block clicking backward	0.85

Sentence comprehension and prediction skills	105	55%	Gender cue activation	0.91

			Verb semantics activation	0.91

Word reading skills	99	40%	Klepel	0.75

			One-minute	0.83

			Maximal speech rate	0.63

			Phonological verbal fluency	0.72


### Participants

We contacted the same 112 native Dutch participants who had previously taken part in the study by Hintz and colleagues (2020) and invited them to take part in the present study. Forty-three of them replied and participated in the self-paced reading experiment. They were paid €6. All participants gave informed consent prior to participation. The ethics board of the Faculty of Social Sciences at Radboud University (Nijmegen, NL) provided ethical approval to conduct the study. Two of the participants were excluded from further analyses (Data pre-processing and analysis section); the remaining 41 participants were on average 22.88 years old (*SD* = 2.8, range = 18–29; 9 male).

### Materials

We selected 25 idiomatic expressions from the Dutch normative database by Hubers et al. (2018, 2019). We embedded the idiomatic expressions in a carrier sentence (see (2) for an example; context in italics, idioms in bold, see Appendix B for all items). Note that the idiom-final noun never occurred in sentence-final position to avoid strategic processing effects and to enable spill-over analyses. This was achieved by adding a semantically neutral word to the sentences.

Each of the 25 target sentences was presented in three conditions: in isolation, preceded by a figuratively biasing context, and preceded by a literally biasing context, amounting to 75 experimental trials containing an idiom. The context sentences were taken from two previous Dutch studies (van Wonderen, Hubers, & Dijkstra, in prep.; van Ginkel, 2019) or created anew:

**Table:** 

(2)	a.	*In deze boekenwinkel heb ik laatst dat mooie boek gevonden*. **Ik tikte hem op de kop** toen.
		Transl.: In this bookstore, I recently found that nice book. I made a good deal that time.
	*b*.	*Die hond heeft laatst mijn schoenen kapotgebeten*. **Ik tikte hem op de kop** toen.
		Transl.: That dog recently bit my shoes to pieces. I tapped him on the head that time.

In addition to the experimental materials, we created 75 non-idiomatic filler items. Twenty-five of which were preceded by a context. Thus, there was an even number of trials with and without context in the experiment. Finally, we created 30 comprehension questions that followed 20% of the experimental and filler trials, which were included to ensure that participants kept focus.

All 50 experimental and 25 filler trials with preceding contexts were tested for plausibility in a rating study conducted via the internet (within the *Pavlovia* web environment, Peirce et al., 2019), involving 56 Dutch native speakers who were paid €4 for participation. These participants did not take part in the main experiment or the study by Hintz et al. (2020). Participants were asked to judge how well the second sentence (e.g., containing the ambiguous idiom) followed-up on the figuratively or literally biasing context (Dutch: ‘Hoe goed volgt de tweede zin op de eerste?’). They responded to the question by selecting a number on a 7-point Likert scale (ranging from 1, not well at all, to 7, very well). The mean plausibility rating for the fifty experimental context-target sentence pairs was 4.37 (*SD* = 1.29, range: 1.47 to 6.17). The 25 trials with figuratively biasing contexts had an average plausibility rating of *M* = 5.24 (*SD* = 0.78, range: 3.17 to 6.17); average plausibility rating of the 25 literally biasing contexts was *M* = 3.50 (*SD* = 1.10, range: 1.74 to 6.00).

### Procedure

The experiment was programmed in *jsPsych* (de Leeuw, 2015) within the *Pavlovia* web environment (Peirce et al., 2019), and run via the internet in participants’ browser. We created an experimental list by shuffling the 75 filler items with the list of 75 experimental items. The 75 experimental items consisted of the 25 sentences with ambiguous idiomatic expressions, presented in the three context conditions. We pseudo-randomized the order of the 150 trials and controlled that there were minimally 10 trials in between two versions of the same idiom. Finally, we created five additional versions of that list by counter-balancing the order of context conditions for the experimental trials. That is, the lists varied in the order in which the context versions of a given idiom were presented (e.g., neutral first, figurative context second, literal context third). The participants were assigned to one of the six experimental lists. They consented to taking part by ticking off a designated box. Participants were instructed to read the sentences silently as fast as possible while still being able to comprehend their contents.

Context sentences were presented for the participant to read in one instance. There was no time limit. Participants initiated the presentation of the target sentence by pressing the enter key. After an interval of 500 ms, a fixation cross appeared for 500 ms, followed by the presentation of the first word. Participants advanced to the next word by pressing the space bar. Reaction time for each word was calculated as the difference between word presentation and button press. Content questions were presented immediately after the last word in a sentence. Participants responded to the question by pressing the keys J (yes-response) and N (no-response). After thirty trials, participants could take a break. The inter-trial interval was 500 ms.

### Data pre-processing and analysis

We used *R* (version 3.5.1, R Core Team, 2018), and the libraries *lme4* (Bates et al., 2015), *lmerTest* (Kuznetsova, Brockhoff, & Christensen, 2017), *effects* (Fox, 2003), *ggplot2* (Wickham, 2016), and *performance* (Lüdecke et al., 2020), to pre-process and analyse the data. Two participants were excluded, because their accuracy on the comprehension questions was lower than 75% (65% and 71%), while all other participants scored substantially higher (*M* = 94,77%; *SD* = 4.23). Data cleaning for the remaining 41 participants was performed on the idiom-final nouns and spill-over words separately. In line with previous studies (Marsden et al., 2018; Prasad & Linzen, 2018), we excluded words with reading times shorter than 100 ms and larger than 2000 ms from further analysis. This led to the exclusion of less than 1% of the data in both analyses. Note that the following analysis was conducted on the basis of the first encounter of an idiom (in one of the three conditions). For an analysis of the full dataset, we refer the reader to Appendix C.

In separate models, we analysed the cleaned reading times of the idiom-final and spill-over words using linear mixed effects regression analyses. The reading times were log-transformed to correct for a right skew in the data. The three-level factor *context* (no context, figuratively biasing context, literally biasing context) was coded using simple contrast coding (UCLA Statistical Consulting Group, 2011). With simple contrast coding, the reference level is always coded as –1/3, and the level that it is compared to is coded as 2/3. This way of coding is similar to treatment contrast coding, but has the advantage that the intercept corresponds to the grand mean instead of corresponding to the mean of the reference level. Moreover, factors outside of interactions can be interpreted as main effects. As continuous predictor variables, we included linguistic knowledge, visual working memory, processing speed, non-verbal IQ, word reading skills, sentence comprehension and prediction skills, as well as length (number of letters) and frequency (Keuleers et al., 2010) of the idiom-final noun/spill-over word and the idiom’s transparency rating (Hubers et al., 2018, 2019). All participant-related and item-related predictors were mean-centred and standardized. We included random intercepts for items and participants. Adding any type of random slopes to the model resulted in overfit.

## Results

The average reading times and standard deviations of the idiom-final nouns and spill-over words per context are presented in ***Table 2***. Differences in reading times between both contexts were very small for both the idiom-final nouns and the spill-over words. Similarly, the differences between both context conditions and the no-context condition were small.

**Table 2:** Average reading times and standard deviations (ms) by context for the idiom final word and the spill-over word.


CONTEXT	IDIOM FINAL NOUN		SPILL-OVER WORD	

	MEAN	SD	MEAN	SD

None	364.93	178.44	418.97	209.46

Figuratively biasing	359.80	155.94	419.23	207.98

Literally biasing	356.45	155.28	414.28	189.95


**Figure 2:**
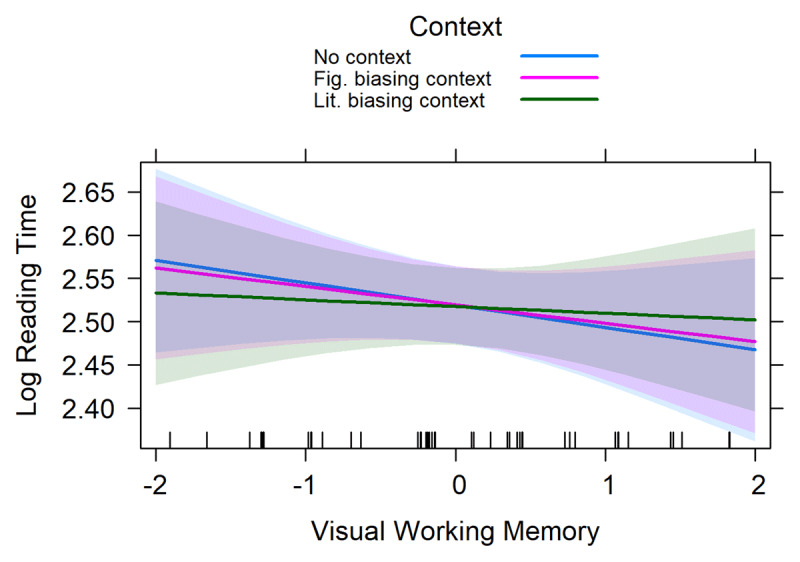
The interaction between Context and Visual working memory for the idiom-final word. The error bands represent the 95% confidence interval.

**Figure 3:**
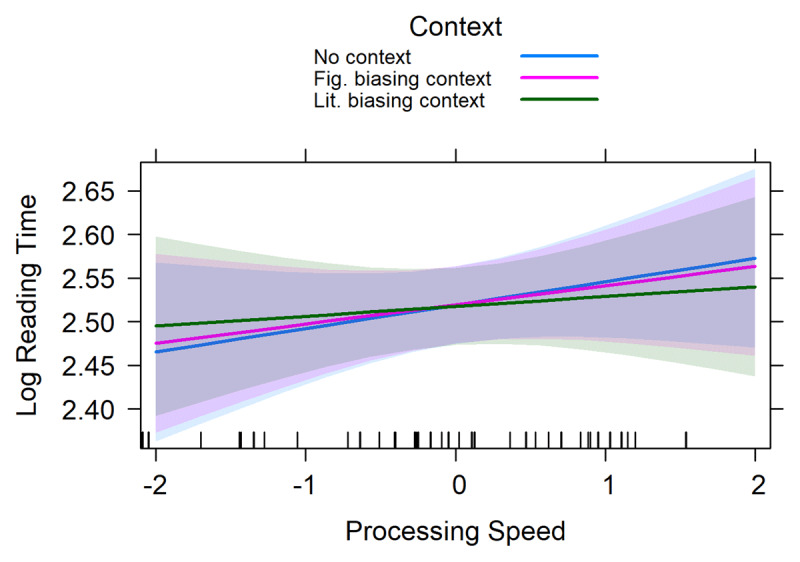
The interaction between Context and Visual working memory for the idiom-final word. The error bands represent the 95% confidence interval.

The results of the linear mixed effects regression analysis on the reading times of the idiom-final noun are presented in ***Table 3***. This analysis revealed a significant main effect of Word reading as well as two interactions: one between *context* and Visual working memory and one between *context* and Processing speed. The main effect of Word reading was negative, suggesting that participants with better word reading skills read the idiom-final nouns faster than participants with lower word reading skills. The two interactions are visualized in ***Figures 2*** and ***3***. ***Figure 2*** suggests that participants with high visual working memory capacity were slower at reading idiom-final nouns in the literally biasing context compared to both neutral and figuratively biasing context conditions. ***Figure 3*** suggests that participants with high non-verbal processing speed abilities (i.e., lower RTs) were faster at reading idiom-final nouns in the literally biasing context compared to the neutral condition.

**Table 3:** Idiom-final noun regression model with logged RTs as dependent variable (the no-context condition as the reference category).


FIXED EFFECTS	*ß* (SE)	*T*	*P*

Intercept	2.4830 (0.0567)	43.768	<0.001***

Fig. biasing context (FBC)	0.0003 (0.0068)	0.049	0.961

Lit. biasing context (LBC)	–0.0015 (0.0068)	–0.216	0.829

Linguistic knowledge	–0.0403 (0.025)	–1.610	0.117

FBC × Ling. knowledge	0.0089 (0.0086)	1.032	0.302

LBC × Ling. knowledge	0.0101 (0.0085)	1.188	0.235

Visual working memory (WM)	–0.0183 (0.0241)	–0.758	0.454

FBC × Visual WM	0.0045 (0.0083)	0.540	0.589

LBC × Visual WM	0.0181 (0.0082)	2.195	0.028*

Processing speed	0.0201 (0.0232)	0.865	0.393

FBC × Processing speed	–0.0048 (0.0078)	–0.622	0.534

LBC × Processing speed	–0.0155 (0.0078)	–1.997	0.046*

Non-verbal IQ	0.0516 (0.0293)	1.763	0.087.

FBC × Non-verbal IQ	–0.0056 (0.0101)	–0.554	0.580

LBC × Non-verbal IQ	–0.0078 (0.0101)	–0.779	0.436

Word reading	–0.0623 (0.0221)	–2.821	0.008**

FBC × Word reading	0.0001 (0.0076)	0.009	0.993

LBC × Word reading	0.0115 (0.0075)	1.527	0.127

Sentence compr. & pred. (SPC)	–0.0076 (0.0244)	–0.312	0.757

FBC × SPC	–0.0014 (0.0082)	–0.169	0.866

LBC × SPC	–0.0020 (0.0082)	–0.239	0.811

Idiom transparency	–0.010019 (0.01)	–1.196	0.245

Idiom final noun frequency	0.0023 (0.0115)	0.204	0.840

Idiom final noun length	0.0065 (0.0089)	0.733	0.472

**RANDOM EFFECTS**	**VARIANCE**	**SD**	

Participant	0.0164	0.128	

Item	0.0019	0.043	

Residual	0.0078	0.088	


The results of the linear mixed effects regression analysis of the spill-over word reading times are presented in ***Table 4***. As in the idiom-final noun analysis, a significant main effect of Word reading was observed, indicating that participants with better word reading skills were faster at reading the spill-over word than participants with poorer word reading skills. No main effect of *context* or any interactions involving *context* were observed.

**Table 4:** Spill-over word regression model with logged RTs as the dependent variable (with the no-context condition as the reference category).


FIXED EFFECTS	*ß* (SE)	*T*	*P*

Intercept	2.5070 (0.1409)	17.794	<0.001***

Fig. biasing context (FBC)	0.0035 (0.0086)	0.403	0.687

Lit. biasing context (LBC)	0.0031 (0.0086)	0.360	0.719

Linguistic knowledge	–0.0400 (0.0244)	–1.638	0.111

FBC × Ling. knowledge	0.0002 (0.0107)	0.023	0.982

LBC × Ling. knowledge	0.0097 (0.0107)	0.908	0.364

Visual working memory (WM)	–0.0321 (0.0235)	–1.364	0.182

FBC × Visual WM	0.0002 (0.0104)	0.016	0.987

LBC × Visual WM	0.0124 (0.0103)	1.203	0.229

Processing speed	0.0248 (0.0226)	1.098	0.280

FBC × Processing speed	–0.0013 (0.0097)	–0.138	0.890

LBC × Processing speed	–0.0101 (0.0097)	–1.044	0.297

Non-verbal IQ	0.0525 (0.0285)	1.840	0.075.

FBC × Non-verbal IQ	0.0015 (0.0126)	0.117	0.907

LBC × Non-verbal IQ	0.0035 (0.0126)	0.274	0.784

Word reading	–0.0591 (0.0215)	–2.746	0.010**

FBC × Word reading	–0.0027 (0.0095)	–0.281	0.779

LBC × Word reading	–0.0048 (0.0095)	–0.501	0.617

Sentence compr. & pred. (SPC)	–0.0179 (0.0238)	–0.754	0.456

FBC × SPC	–0.0040 (0.0103)	–0.390	0.697

LBC × SPC	0.0003 (0.0103)	0.025	0.980

Idiom transparency	–0.0166 (0.0110)	–1.508	0.147

Spill-over word frequency	0.0019 (0.0246)	0.077	0.939

Spill-over word length	0.0105 (0.0071)	1.492	0.151

**RANDOM EFFECTS**	**VARIANCE**	**SD**	

Participant	0.0154	0.124	

Item	0.0025	0.050	

Residual	0.0121	0.110	


## Data discussion

In contrast to our hypotheses, we observed no main effect of context. However, as one would expect, we observed that individuals with better word reading abilities read idiom-final and spill-over words faster (in all three context conditions) than individuals with poorer word-reading skills. These effects were seen in both types of analyses we conducted—based on the first encounter of a given item (main analysis) and based on the full dataset (see Appendix C). While the fact that better reading ability led to overall faster reading of idiom-final and spill-over words is not necessarily a novel finding, it does demonstrate that the present self-paced reading experiment (conducted via the internet) indeed picked up individual differences as measured in a different study, which was conducted almost one year before the present experiment (Hintz et al., 2020).

In the analysis based on the first encounter of an item, we additionally observed evidence for modulatory influences of visual working memory and non-verbal processing speed on reading idiom-final targets in the literally biasing context (but not on no-context and figuratively biasing context) condition: Individuals with better processing speed abilities read idiom-final nouns in that condition faster than individuals with worse processing speed abilities, relative to the neutral condition. Moreover, readers with higher visual working memory capacities had longer RTs for idiom-final nouns than readers with lower capacities. These effects suggest that participants were differentially affected by idioms presented in contexts that biased the literal interpretation of an idiom’s constituent words. One possible linking hypothesis for this data pattern is that individuals with better processing speed abilities might have been able to link the preceding (‘deidiomatizing’) context to the unfolding target sentence more quickly than individuals with lower processing speed abilities could. They were thus faster and more efficient at switching off the idiomatic meaning, which led to faster target processing. The inhibitory effect of visual working memory on target word processing is in contrast to our hypotheses, which predicted that readers with larger visual working memory capacity should remember and use preceding contexts more efficiently than readers with lower capacities, leading to faster target word processing. The opposite was the case and we cannot offer a good account for this finding. Future users of the data resource could explore this finding in more detail and, for example, conduct analyses where multiple individual-differences predictors (among others, visual working memory) interact.

In general, from a statistics point of view, future research could explore different ways of analyzing the data. One may, for example, fit regression models with different random-effect structures than the one used in the present model. Similarly, further work could address whether and if so, how, repeating the same idiom twice within participants affected their processing. That is, while the main effects of Word reading were consistent across both types of analyses, the two interactions involving the literally biasing context condition discussed above were not observed when item repetitions were included. Instead, we saw effects of non-verbal IQ and linguistic knowledge (in interaction with the literally biasing context condition; see Appendix C, for a more detailed description).

In sum, the present data resource offers many exciting avenues for conducting additional exploratory and/or targeted analyses, especially when linked to the dataset provided by Hintz et al. (2020). We hope that researchers make use of it to advance the field of idiom processing and/or individual differences. The data can be accessed at *https://hdl.handle.net/1839/5005965b-f11f-4c7a-a82d-ad6f6b6e58d4*. Interested researchers need to create a free account with the Archive of the Max Planck Institute for Psycholinguistics providing a user name, email address, their full name and affiliation. Alternatively, in case their institution is part of one of the supported Identity Federations (Shibboleth), which is the case for many academic/research institutions, interested individuals may simply use their own institutional account to log in. Use of the data is confined to academic purposes.

## Data Accessibility Statement

The archived materials, analysis scripts, logfiles and results can be found here: *https://hdl.handle.net/1839/5005965b-f11f-4c7a-a82d-ad6f6b6e58d4*.

## Additional Files

The additional files for this article can be found as follows:

10.5334/joc.183.s1 Appendix A. Descriptive statistics and reliability measures of each individual-differences test.

10.5334/joc.183.s2 Appendix B. Materials.

10.5334/joc.183.s3 Appendix C. Analysis based on full dataset (including item repetitions within a participant).

## Footnotes

As for the present data, all data collected by Hintz et al. (2020) are publicly available. Thus, if researchers would like to include additional/different individual-differences variables, they may download the data from UK Data Archive: *https://reshare.ukdataservice.ac.uk/854399/*.
