# Supplementary material for: Exploring Individual Differences in Recognizing Idiomatic Expressions in Context
Source: J Cogn. 2021 Aug 12;4(1):37. doi: 10.5334/joc.183 (PMC8362631; doi:10.5334/joc.183)
Supplement: Appendix A. — Descriptive statistics and reliability measures of each individual-differences test. [file joc-4-1-183-s1.pdf]

Appendix A - Descriptive statistics and reliability measures of each individual-differences test

Table A1: Descriptive statistics and reliability measures taken from Hintz et al. (2020).

Domain	Test	N	Mean (SD)	Range	Skewness [a]	Kurtosis [a]	Internal consistency	Retest reliability [f]	Errors (%)	Outliers (%)	
										S1	S2
Linguistic Knowledge	Peabody picture vocabulary test	112	56 (25)	0 – 95	-0.43	-0.79	0.96 [b]	0.91	-	-	
	Spelling test	112	0.56 (0.18)	0.1 – 0.93	-0.43	-0.37	0.83 [c]	0.85	-	-	
	Dutch author recognition test	112	0.2 (0.12)	-0.03 – 0.6	0.62	0.47	0.93 [c]	0.95	-	-	
	Idiom recognition test	112	0.76 (0.13)	0.4 – 1	-0.33	0.03	0.53 [c]	0.78	-	-	
	Prescriptive grammar test	112	0.69 (0.13)	0.4 – 1	0.04	-0.65	0.74 [c]	0.86	-	-	
General cognitive skills	Auditory simple RT test	112	Log: 2.35 (0.08)<br>Raw: 235 (48)	Log: 2.2 – 2.65<br>Raw: 160 – 459	-1.36 [d]	3.1 [d]	0.9 [de]	0.59 [d]	-	0.85	1.21 [d]
	Auditory choice RT test	112	Log: 2.6 (0.09)<br>Raw: 417 (100)	Log: 2.41 – 2.86<br>Raw: 263 – 799	-0.6 [d]	0.15 [d]	0.96 [de]	0.76 [d]	3.75	0.81	0.49 [d]
	Letter comparison test	107	Log: 3.02 (0.08)<br>Raw: 1167 (251)	Log: 2.86 – 3.28<br>Raw: 748 – 2044	0.65 [d]	0.47 [d]	0.89 [de]	0.83 [d]	6.89	2.01	0.08 [d]
	Visual simple RT test	112	Log: 2.37 (0.05)<br>Raw: 244 (33)	Log: 2.24 – 2.55<br>Raw: 179 – 358	-0.54 [d]	0.51 [d]	0.86 [de]	0.58 [d]	-	0.49	1.88 [d]
	Visual choice RT test	112	Log: 2.62 (0.07)<br>Raw: 439 (90)	Log: 2.5 – 2.86<br>Raw: 321 – 822	0.88 [d]	0.73 [d]	0.95 [de]	0.78 [d]	4.13	0.19	0.49 [d]
Visual working memory	Corsi block clicking test forward	111	8 (2)	3 – 12	-0.08	0.25	0.53 [c]	0.39	-	-	
	Corsi block clicking test backward	108	7 (2)	3 – 12	-0.04	-0.15	0.71 [c]	0.49	-	-	
Word reading skills	Verbal fluency phonology	112	16 (4)	3 – 30	0.15	0.55	-	0.71	-	-	
	Maximal speech rate	106	Log: 3.60 (0.09)<br>Raw: 4028 (854)	Log: 3.39 – 3.82<br>Raw: 2458 – 6650	-0.24	-0.07	-	0.88	-	-	
	One-minute test	111	90 (14)	56 – 116	-0.12	-0.57	0.46 [c]	0.79	-	-	
	Klepel test	111	63 (12)	34 – 107	0.26	0.6	0.88 [c]	0.88	-	-	
Sentence comprehension and prediction skills	Gender cue activation during sentence comprehension	105	-588 (655)	-1674 – 940	0.45	-0.95	0.88 [e]	0.88	1.6	0.25	0.82
	Verb semantics activation during sentence comprehension	112	-742 (673)	-1701 – 1041	0.62	-0.72	0.86 [e]	0.76	0.96	0.65	0.68

See Usage Notes section for missing values in column 'N'.

Values in S1 and S2 columns indicate the percentage of trials replaced during Stage 1 (trimming) and Stage 2 (outlier replacement) in the pre-processing pipeline.

[a] Calculated based on aggregated performance indicators.

[b] Internal consistency was calculated as Guttman's Lambda-2 coefficient.

[c] Internal consistency was calculated by adjusting split-half (odd–even) correlations with the Spearman-Brown prophecy formula.

[d] Calculated based on log-transformed values.

[e] Internal consistency was calculated as intra-class correlation coefficient 2 using the 'psychometric' package in R.

[f] *Test-retest reliability was operationalized as two-tailed Pearson's correlation between performance on test days 1 and 2*
