# Supplementary material for: Exploring Individual Differences in Recognizing Idiomatic Expressions in Context
Source: J Cogn. 2021 Aug 12;4(1):37. doi: 10.5334/joc.183 (PMC8362631; doi:10.5334/joc.183)
Supplement: Appendix B. — Materials. [file joc-4-1-183-s2.pdf]

Appendix B – Materials

Experimental items

**Figurative context**	**Literal context**	**Target SPR idiom** *(both lit./fig. translation)*	**Associated question**	**Mean Rating of plausibility** **Figurative pair**	**Literal pair**
De oplichter heeft ons geld afgetroggeld.<br><br>*The swindler took money from us.*	De tuinman heeft het gazon net gezaaid, dus mochten we er niet overheen lopen.<br><br>*The gardener had just sown the lawn, so we were not allowed to walk on it.*	Hij leidde ons om de tuin gisteren.<br><br>*He led us around the garden yesterday.*<br><br>*He fooled us yesterday.*	*[only in lit. cond.]:*<br><br>Leidde de huismeester ons om de tuin? (nee)<br><br>*Did the housekeeper lead us around the garden? (no)*	5.36	3.5
Herman kan niet tegen het zien van bloed.<br><br>*Herman cannot stand the sight of blood.*	Met eten kun je die parkiet altijd lokken.<br><br>*You can always lure the parakeet with food.*	Hij ging meteen van zijn stokje de vorige keer.<br><br>*He immediately went off his pole last time.*<br><br>*He immediately fainted last time.*	*[only in fig. cond.]:*<br><br>Kan Herman tegen het zien van bloed? (nee)<br><br>*Can Herman stand the sight of blood? (no)*	6.16	3.46
De leraar was snel boos.<br><br>*The teacher was quick to anger.*	De kaars was snel opgebrand.<br><br>*The candle burned out quickly.*	Hij had een kort lontje blijkbaar.<br><br>*It had a short fuse, apparently.*<br><br>*He was temperamental, apparently.*	*[only in neutral cond.]:*<br><br>Had hij een kort lontje? (ja)<br><br>*Did he have a short fuse? (yes)*	5.94	4.13

Zijn humeur was erg slecht.<br><br>*His mood was very bad.*	Zijn pantoffels waren te groot.<br><br>*His house shoes were too large.*	Hij schoot steeds uit zijn slof vanochtend.<br><br>*He kept falling out of his slipper this morning.*<br><br>*He kept going off the rails this morning.*	*[only in lit. cond.]:*<br><br>Waren zijn pantoffels te groot? (ja)<br><br>*Were his slippers too big? (yes)*	5.90	3.25
Het meisje wilde haar ouders niet vertellen waarom ze na moest blijven.<br><br>*The girl did not want to tell her parents why she was kept in detention after school.*	Moeder repareerde de gaten in de truien van haar kinderen.<br><br>*Mother repaired the holes in her children's jumpers.*	Ze speldde hun iets op de mouw toen.<br><br>*She pinned something on their sleeve then.*<br><br>*She lied then.*		3.65	2.83
Eva is de laatste tijd depressief.<br><br>*Eva has been depressed lately.*	Tijdens het spelen op de boerderij viel een van de kinderen naar beneden.<br><br>*While playing at the farm, one of the children fell down.*	Ze zit diep in de put nu.<br><br>*She is deep in the pit now.*<br><br>*She is in a dark place now.*		5.52	3.71
De agent was in gesprek met de verdachte.	De tandarts controleerde de pijnlijke kies van de man.	Hij voelde hem aan de tand gisteren.<br><br>*He checked his tooth*	*[only in fig. cond.]:*<br><br>Was de agent in gesprek met zijn baas? (nee)	5.39	2.88

The officer was talking to the suspect.	*The dentist controlled the man's painful tooth.*	*yesterday.*<br><br>*He questioned him yesterday.*	*Was the officer talking to his superior? (no)*		
In deze boekenwinkel heb ik laatst dat ene mooie boek gevonden.<br><br>*I found that one beautiful book in this bookstore the other day.*	De hond van mijn ouders heeft laatst mijn schoenen kapot gebeten.<br><br>*My parents' dog recently bit my shoes off.*	Ik tikte hem op de kop toen.<br><br>*I tapped him on the head at the time.*<br><br>*I made a good deal at the time.*	*[only in fig. cond.]:*<br><br>Was het boek duur? (nee)<br><br>*Was the book expensive? (no)*	4.55	4.33
Zijn moeder bemoeit zich erg met de keuzes van haar kinderen.<br><br>*His mother is very meddlesome in her children's choices.*	De peuter speelt tijdens het ontbijt altijd met haar eten.<br><br>*The toddler always plays with her food during breakfast.*	Ze heeft een vinger in de pap helaas.<br><br>*She has a finger in the mush, unfortunately.*<br><br>*She is intrusive, unfortunately.*	*[only in neutral cond.]:*<br><br>Heeft ze een vinger in de pap? (ja)<br><br>*Does she have a finger in the mush? (yes)*<br><br>*Is she intrusive? (yes)*	4.16	2.71
Jesse doet totaal niet zijn best op het werk.<br><br>*Jesse is not doing his best at work at all.*	De kleuter heeft een vieze gewoonte.<br><br>*The toddler has a nasty habit.*	Hij eet uit zijn neus soms.<br><br>*He eats out of his nose sometimes.*<br><br>*He is being lazy sometimes.*		5.03	5.33
Die handelaar	Het was nog erg donker	Hij liep tegen de lamp	*[only in lit. cond.]:*	5.07	4.13

verkocht gestolen fietsen tegen hoge prijzen.<br><br>*That dealer sold stolen bicycles at high prices.*	in de woonkamer omdat Geert het licht niet aangezet had.<br><br>*It was still very dark in the living room because Geert had not turned on the light.*	uiteindelijk.<br><br>He eventually ran into the lamp.<br><br>*He eventually was caught.*	Was het donker in de woonkamer? (ja)<br><br>*Was it dark in the living room? (yes)*		
De man doet niet lang over het drinken van een fles wijn.<br><br>*The man does not take long to drink a bottle of wine.*	De generaal wilde dat zijn zoon ook het leger in zou gaan.<br><br>*The general wanted his son to join the army too.*	Hij maakte hem soldaat zo snel het kon.<br><br>*He made a soldier out of him as quickly as he could.*<br><br>*He emptied/finished it as quickly as he could.*	*[only in fig. cond.]:*<br><br>Doet de man lang over het drinken van een fles wijn? (nee)<br><br>*Does the man take long to drink a bottle of wine? (no)*	5.16	3.96
In het gezin bleef de zachtaardige man op de achtergrond.<br><br>*In the family, the gentle man remained in the background.*	De man lag in zijn stoel te slapen op de boot.<br><br>*The man was asleep in his seat on the boat.*	Zijn vrouw stond aan het roer op dat moment.<br><br>*His wife was at the helm at the time.*<br><br>*His wife wore the britches at the time.*	*[only in neutral cond.]:*<br><br>Stond zijn dochter aan het roer? (nee)<br><br>*Was his daughter at the helm? (no)*	4.45	6.00
Jan liet Corine vroeger altijd schrikken.<br><br>*Jan used to startle*	Roy had gisteren meel gehaald omdat Lisa wilde gaan bakken.	Zij gaf hem een koekje van eigen deeg nu.<br><br>*She gave him a cookie of*		6.04	2.23

Corine.	*Roy had fetched flour yesterday because Lisa wanted to bake.*	*own dough now.*<br><br>*She gave him a taste of her own medicine now.*		
Wonderkind Jeanne leerde wel drie nieuwe talen tegelijkertijd.<br><br>*Child prodigy Jeanne learned as many as three new languages at the same time.*	Karin moest na het ongeluk een beenamputatie ondergaan.<br><br>*Karin had to undergo a leg amputation after the accident.*	Zij kreeg die onder de knie afgelopen week.<br><br>*She got it/them under her knees.*<br><br>*She mastered it/them last week.*	4.75	2.19
De ballerina deed auditie voor de hoofdrol.<br><br>*The ballerina auditioned for the lead role.*	De vrouw verkocht veel van haar oude spullen.<br><br>*The woman sold many of her old things.*	Ze haalde alles uit de kast vanmiddag.<br><br>*She pulled everything out of the cabinet.*<br><br>*She pulled out all the stops this afternoon.*	6.04	3.77
Omdat veel werknemers moesten overwerken, schakelde de directeur extra personeel in.<br><br>*As many employees had to work overtime, the director called in*	De machinist van de oude stoomtrein opende een uitlaat, want ze gingen wel erg snel.<br><br>*The conductor of the old steam train opened an exhaust, for they were going very fast.*	Dat haalde wat druk van de ketel gelukkig.<br><br>*Fortunately, that took some of the pressure off the cauldron.*<br><br>*Fortunately, that made things a bit less intense.*	6.16	3.77

extra staff.				
De dader werd uiteindelijk niet schuldig bevonden, omdat zijn vrouw hem hielp.<br><br>*The perpetrator was eventually found not guilty because his wife helped him.*	De vrouw van de achtbaan gaf aan hoe lang het jongetje moest zijn om mee te mogen.<br><br>*The woman at the roller coaster indicated how tall the little boy had to be to be allowed on.*	Zij hield hem een hand boven het hoofd helaas.<br><br>*She held a hand over his head, unfortunately.*<br><br>*She protected him, unfortunately.*	5.29	2.03
Stijn en Maartje irriteerden elkaar al de hele dag.<br><br>*Stijn and Maartje had been annoying each other all day.*	Bas en Els zijn concurrenten voor de winst tijdens de hardloopwedstrijd.<br><br>*Bas and Els are competitors for the win in the running competition.*	Uiteindelijk ging zij als eerste door het lint vanochtend.<br><br>*In the end, she was the first to go through the finishing straight this morning.*<br><br>*In the end, she was the first to lose her temper this morning.*	5.17	2.87
De reizigers hadden geen kaartje toen de conducteur hen kwam controleren.<br><br>*The passengers had no ticket when the conductor came to*	Moeder had te veel spullen meegenomen naar het park.<br><br>*Mother had taken too many things to the park.*	Ze vielen door de mand helaas.<br><br>*Unfortunately, they fell through the basket.*<br><br>*Unfortunately, they did not get away with it.*	6.04	2.90

check them.					
Wendy heeft het ontzettend druk.<br><br>*Wendy is very busy.*	Eefje wil graag groter lijken dan ze is.<br><br>*Eefje would like to look bigger than she is.*	Ze loopt op haar tenen sinds vorige week.<br><br>*She has been tiptoeing since last week.*<br><br>*She has been stressed since last week.*		5.04	3.97
De directeur was niet tevreden met het huidige beleid.<br><br>*The director was not satisfied with the current policy.*	De piraat vergistte zich in de kant van het schip waar het anker hoorde.<br><br>*The pirate mistook the side of the ship where the anchor belonged.*	Hij gooide het over een andere boeg gisteren.<br><br>*He threw it over another [ship] bow yesterday.*<br><br>*He changed course yesterday.*	*[only in lit. cond.]:*<br><br>Vergistte de piraat zich? (ja)<br><br>*Was the pirate making a mistake? (yes)*	5.75	3.03
Coen had nooit verwacht dat hij de baan echt zou krijgen.<br><br>*Coen never expected that he would actually get the job.*	De piloot vloog duidelijk lager dan normaal.<br><br>*The pilot was clearly flying lower than normal.*	Hij was in de wolken die dag.<br><br>*He was in the clouds that day.*<br><br>*He was on cloud nine that day.*		3.17	5.94
Teun winkelde erg vaak.	Jorick heeft een naar ongeluk gehad.	Hij had een gat in zijn hand vroeger.		5.63	2.90

Teun shopped a lot.	*Jorick had a bad accident.*	*He used to have a hole in his hand.*<br><br>*He used to spend too much money.*		
Hein was al heel oud.<br><br>*Hein was already very old.*	De klusjesman was erg goed met metaal.<br><br>*The handyman was very good with metal.*	Hij legde het loodje eergisteren.<br><br>*He laid the lead [pipe] yesterday.*<br><br>*He died the day before yesterday.*	5.63	1.74

Filler items

**Context sentence**	**SPR sentence**	**Associated question**
Met mijn vrienden was ik kamperen in Spanje, waar het weer heel aangenaam was.<br><br>*I was camping with my friends in Spain, where the weather was very pleasant.*	We sliepen in de open lucht zonder tent.<br><br>*We slept in the open without a tent.*	Waren we kamperen in Spanje? (ja)<br><br>*Were we camping in Spain? (yes)*
Als je gaat picknicken op het strand moet je wel goed opletten.<br><br>*When you go on a picnic on the beach, you have to be careful.*	Anders zit je eten snel onder het zand.<br><br>*Otherwise, your food will soon be covered in sand.*	Moet je opletten dat je eten nat wordt? (nee)<br><br>*Do you have to be careful not to get your food wet? (no)*
Vanuit haar balkon had Maaike goed zicht op de vechtpartij op straat.<br><br>*From her balcony, Maaike had a good view of the brawl in the street.*	Daarom riep de politie haar op als getuige.<br><br>*That is why the police called her as a witness.*	Had Maaike zicht op een verkeersongeluk? (nee)<br><br>*Did Maaike have a view on a traffic accident? (no)*
Scheiden is altijd lastig, zeker als er kinderen bij betrokken zijn.<br><br>*Divorce is always difficult, especially when children are involved.*	Vaak is er dan ruzie over de voogdij.<br><br>*There are often fights over custody.*	Is er vaak ruzie over wie er voor de kinderen mag zorgen? (ja)<br><br>*Do you often argue about who can take care of the children? (yes)*
Suzanne had al een paar maanden lang last van haar kies toen ze naar de tandarts ging.<br><br>*Suzanne had been suffering from a toothache for a few months when she went to the dentist.*	Ze kreeg een verdoving tegen de pijn.<br><br>*She was given an anaesthetic against the pain.*	Had Suzanne last van haar teen? (nee)<br><br>*Was Suzanne bothered by her toe? (no)*
Naar de bioscoop gaan is heel leuk.	Voor de film begint, haal ik altijd een grote bak popcorn.	Haal ik altijd een grote bak popcorn? (ja)

Going to the cinema is great fun.	*Before the film starts, I always get a big bowl of popcorn.*	*Do I always get a big bowl of popcorn? (Yes)*
Johan en Linda vierden onlangs hun 25-jarig huwelijk.<br><br>*Johan and Linda recently celebrated their 25th wedding anniversary.*	Dus nodigden ze de hele familie uit voor een groot feest.<br><br>*So they invited the whole family to a big party.*	Nodigden Johan en Linda al hun vrienden uit? (nee)<br><br>*Did Johan and Linda invite all their friends? (no)*
Zelf podcasts opnemen is helemaal niet zo goedkoop.<br><br>*Recording your own podcasts isn't that cheap.*	Je moet namelijk heel wat betalen voor een goede microfoon.<br><br>*After all, you have to pay a lot of money for a good microphone.*	Is zelf podcasts opnemen duurder dan verwacht? (ja)<br><br>*Is recording your own podcasts more expensive than you expected? (Yes)*
Ferdy zorgt ervoor dat hij elke ochtend stevig ontbijt.<br><br>*Ferdy makes sure he has a hearty breakfast every morning.*	Vaak is er nauwelijks nog plek op zijn bord.<br><br>*Often there is hardly any room left on his plate.*	
Emma vindt de geur van sigaretten verschrikkelijk.<br><br>*Emma hates the smell of cigarettes.*	De kleren van haar vriendin stinken altijd naar rook.<br><br>*Her friend's clothes always stink of smoke.*	
Tijdens een marathon is het belangrijk om goed te blijven drinken.<br><br>*During a marathon, it is important to keep drinking well.*	Toen de loper door de laatste bocht kwam gaf iemand hem water.<br><br>*When the runner came through the last bend, someone gave him water.*	
Als ik op een lange reis ga, leen ik vaak wat boeken bij de bibliotheek.<br><br>*When I go on a long trip, I often borrow some books from*	Soms lever ik ze niet op tijd in en krijg ik een enorme boete.<br><br>*Sometimes I don't return them on time and get a huge fine.*	

the library.		
Marjolijn vindt gokken heel leuk maar is niet goed in kaartspelen.<br><br>*Marjolijn likes gambling very much but she is not good at playing cards.*	Vorige week verloor ze veel geld tijdens het pokeren.<br><br>*Last week she lost a lot of money playing poker.*	
Ik ging vaak op zaterdagochtend vissen met mijn vader.<br><br>*I often went fishing on Saturday morning with my father.*	We namen altijd een emmer wormen mee als aas.<br><br>*We always took a bucket of worms as bait.*	
Jagers moeten er op letten dat ze niet per ongeluk elkaar neerschieten.<br><br>*Hunters have to take care not to accidentally shoot each other.*	Daarom dragen ze vaak een oranje vest.<br><br>*That's why they often wear an orange vest.*	
Petra is erg blij dat ze tijdens de schaatswedstrijd derde geworden is.<br><br>*Petra is very happy that she came third in the speed skating competition.*	Zij wilde zo graag een plek op het podium.<br><br>*She so badly wanted a place on the podium.*	
Bij de NS zijn er momenteel erg vaak werkzaamheden.<br><br>*At the moment, there are a lot of works on the NS [Dutch National Railway Company].*	Tussen Arnhem en Den Bosch rijden nu alleen maar bussen.<br><br>*Between Arnhem and Den Bosch only buses are running at the moment.*	
Studentenhuizen staan bekend om hun niet al te hygiënische toiletten.<br><br>*Student houses are known for*	Als je die schoon wilt maken, kun je beter handschoenen dragen.<br><br>*If you want to clean them,*	

their not very hygienic toilets.	*you'd better wear gloves.*	
Mijn ouders waren erg blij toen mijn zus eindelijk ging trouwen.<br><br>*My parents were very happy when my sister finally got married.*	Mijn moeder moest zelfs huilen tijdens de ceremonie.<br><br>*My mother even cried during the ceremony.*	
Afgelopen zaterdag heb ik een tweedehands televisie gekocht, maar ik kan niet van zender wisselen.<br><br>*Last Saturday I bought a second-hand television, but I can't change the channel.*	Wat er niet bij zat was een afstandsbediening.<br><br>*What it didn't come with was a remote control/*	
Een van de boksers viel uiteindelijk op de mat.<br><br>*One of the boxers finally fell on the mat.*	De scheidsrechter riep de ander uit tot winnaar.<br><br>*The referee declared the other one the winner.*	
Reizen met het vliegtuig duurt tegenwoordig veel langer, omdat iedereen bang is voor terrorisme.<br><br>*Travelling by plane takes much longer nowadays, because everyone is afraid of terrorism.*	Op vliegvelden staat er altijd een lange rij voor de douane.<br><br>*At airports, there is always a long queue for customs.*	
Op een boerderij wonen heeft ook wel nadelen.<br><br>*Living on a farm also has its disadvantages.*	Elke ochtend werd ik vroeg wakker door het gekraai van de haan.<br><br>*Every morning, I was woken up early by the crowing of the rooster.*	
Heb je afgelopen zaterdag de voetbalwedstrijd gezien?	Die spits scoorde echt een hele mooie goal.	

Did you see the football match last Saturday?	*That striker scored a really nice goal.*	
Joeri gaat in de ochtendspits soms op de fiets naar zijn werk.<br><br>*In the morning rush hour, Joeri sometimes goes to work by bike.*	Dat gaat vaak zelfs sneller dan met de auto.<br><br>*That is often even faster than going by car.*	
	Soms schrikken de kinderen wel van het gebrul van de leeuw.<br><br>*Sometimes the lion's roar scares the children.*	Schrikken de kinderen van het gebrul van de tijger? (nee)<br><br>*Does the roaring of the tiger frighten the children? (no)*
	Hij is me nog aardig wat geld schuldig.<br><br>*He still owes me a lot of money.*	Ben ik hem nog aardig wat geld verschuldigd? (nee)<br><br>*Do I owe him a lot of money? (no)*
	Hij heeft duidelijk nog niet veel ervaring.<br><br>*He clearly doesn't have much experience yet.*	Heeft deze medewerker weinig ervaring? (ja)<br><br>*Does this employee have little experience? (yes)*
	Planten in huis maken het meteen gezelliger.<br><br>*Plants in the house make it cosier.*	Maken planten het huis gezelliger? (ja)<br><br>*Do plants make the house cosier? (yes)*
	Op het einde word je wel beloond met een fantastisch uitzicht.<br><br>*At the end, you are rewarded with a fantastic view.*	Word je op het einde beloond met lekker eten? (nee)<br><br>*Are you rewarded with good food at the end? (no)*
	Daar werkte ik namelijk achter de kassa.<br><br>*The thing is, I worked there*	Werkte ik achter de kassa? (ja)<br><br>*Was I working behind the*

	behind the cash register.	*counter? (Yes)*
	Hij wil heel graag een grotere tafel.<br><br>*He would like a bigger table.*	Heeft Martijn meubels nodig voor zijn slaapkamer? (nee)<br><br>*Does Martin need furniture for his bedroom? (no)*
	De vrouw leefde al jaren alleen.<br><br>*The woman had been living alone for years.*	Leefde de vrouw alleen? (ja)<br><br>*Did the woman live alone? (Yes)*
	Hij is me nog aardig wat geld schuldig.<br><br>*He still owes me a lot of money.*	Ben ik hem nog aardig wat geld verschuldigd? (nee)<br><br>*Do I owe him a lot of money? (no)*
	Hij moest toch lachen uiteindelijk.<br><br>*He had to laugh in the end.*	
	Voor al haar documenten wilde ze een nieuwe laptop.<br><br>*She wanted a new laptop for all her documents.*	
	Dat wordt waarschijnlijk een flink litteken.<br><br>*That will probably be quite a scar.*	
	Na een paar uur wilde zij nog steeds niet het zwembad uit.<br><br>*After a few hours, she still did not want to leave the pool.*	
	Uiteindelijk besloten we te stoppen om te vragen naar de weg.	

	Finally, we decided to stop and ask for directions.	
	Vleermuizen vind ik erg fascinerende dieren.<br><br>*I find bats very fascinating animals.*	
	Gelukkig is hij nu weer gemaakt door de reparateur.<br><br>*Fortunately, it has been repaired by the repairman.*	
	Toen ze de top bereikten, werden ze overweldigd door het uitzicht.<br><br>*When they reached the top, they were overwhelmed by the view.*	
	Als ze klaar is, is ze helemaal nat van het zweet.<br><br>*When she's finished, she's all wet with sweat*<br>.	
	Dat is overigens altijd al zo geweest.<br><br>*It has always been like that, by the way.*	
	Buiten bouwden ze een grote sneeuwpop.<br><br>*Outside they built a big snowman.*	
	Zij willen graag een groter huis.<br><br>*They would like a bigger house.*	

	Om mij te feliciteren, stuurde ze me nog wel een kaartje.<br><br>*To congratulate me, they sent me a card.*	
	Het is altijd moeilijk om een goed cadeau te bedenken.<br><br>*It is always difficult to think of a good gift.*	
	Die paar weken rust hebben hem zeker goed gedaan.<br><br>*Those few weeks of rest have certainly done him good.*	
	Tijdens de Vierdaagse is er behoorlijk veel lawaai.<br><br>*During the Four Days Marches there is a lot of noise.*	
	Met een theoretische opleiding vind je tegenwoordig toch geen baan.<br><br>*You can't find a job with a theoretical education these days anyway.*	
	Ik zou de hele week pizza kunnen eten.<br><br>*I could eat pizza all week.*	
	Zij had veel te lang niet meer geschaatst.<br><br>*She had not skated for far too long.*	
	Gelukkig zie je de vlek niet zo goed op mijn donkere trui.	

	Luckily you can't see the stain so well on my dark jumper.	
	Op zondagavond kijkt ze altijd naar dat programma van Lubach.<br><br>*She always watches that show of Lubach [Dutch late night show host] on Sunday evening.*	
	Laatst heb ik koeien zien lopen op straat.<br><br>*The other day, I saw cows walking on the street.*	
	Naast kleding zijn we ook op zoek naar mooie handtassen.<br><br>*Apart from clothes, we are also looking for beautiful handbags.*	
	Ze gaat echt heel graag zwemmen.<br><br>*She really likes to go swimming.*	
	Ik heb heel wat zaadjes geplant in mijn moestuin.<br><br>*I planted a lot of seeds in my vegetable garden.*	
	We hadden echt veel te veel gegeten.<br><br>*We had really eaten too much.*	
	Ik sta vaak een lange tijd doelloos naar mezelf te staren in de spiegel.<br><br>*I often spend a long time*	

	staring at myself aimlessly in the mirror.	
	Hij kookt nu vijf keer per week.<br><br>*He cooks five times a week now.*	
	Ze was zelden zo gelukkig geweest.<br><br>*She had rarely been so happy.*	
	Hij werd gelukkig snel in veiligheid gebracht door de badmeester.<br><br>*Fortunately, he was quickly taken to safety by the lifeguard.*	
	Sindsdien draagt hij altijd een helm.<br><br>*Since then, he always wears a helmet.*	
	Ik had mijn hond het liefst meegenomen op reis.<br><br>*I would have preferred to take my dog with me on a trip.*	
	Je kunt dit natuurlijk ook uitbesteden aan een verhuisbedrijf.<br><br>*Of course, you can also outsource this to a removal company.*	
	Ze pakte het vliegtuig vorige week.<br><br>*She took the plane last week.*	

	Ze vroeg de dokter om een nieuwe afspraak.<br><br>*She asked the doctor for a new appointment.*	
	Bij schrijven is het begin altijd het moeilijkst.<br><br>*When writing, the beginning is always the hardest.*	
	Bach is mijn favoriete componist tegenwoordig.<br><br>*Bach is my favourite composer these days.*	
	Hij moest daar erg om lachen.<br><br>*He had to laugh about it.*	
	Waar mogelijk neemt ze liever de lift.<br><br>*Whenever possible, she prefers to take the lift.*	
	De kinderen renden weg zo snel als ze konden.<br><br>*The children ran away as fast as they could.*	
	Even later hoorde ze een verschrikkelijke knal.<br><br>*A little later she heard a terribly loud bang.*	
	Hij werkte namelijk in de kerk als pastoor.<br><br>*He was working in the church as a priest.*	
