# Supplementary material for: Exploring Individual Differences in Recognizing Idiomatic Expressions in Context
Source: J Cogn. 2021 Aug 12;4(1):37. doi: 10.5334/joc.183 (PMC8362631; doi:10.5334/joc.183)
Supplement: Appendix C. — Analysis based on full dataset (including item repetitions within a participant). [file joc-4-1-183-s3.pdf]

Appendix C – Analysis based on full dataset (including item repetitions within a participant)

The average reading times and standard deviations of the idiom-final nouns and spill-over words per context are presented in Table C1. Differences in reading times between contexts were very small for both the idiom-final nouns and the spill-over words.

Table C1: Average reading times and standard deviations (ms) by context for the idiom final word and the spill-over word.

Context	Idiom final noun		Spill-over word	
	Mean	SD	Mean	SD
None	304.73	143.55	355.39	182.74
Figuratively biasing	302.89	138.10	358.24	184.04
Literally biasing	301.91	129.62	355.23	170.72

Figure C1 presents the mean differences between the figuratively biasing context and the no context condition (left panels) and the mean differences between the literally biasing context and the no context condition (right panels) per participants. Positive values indicate contextual facilitation. The figure shows that participants were differently affected by our context manipulation.

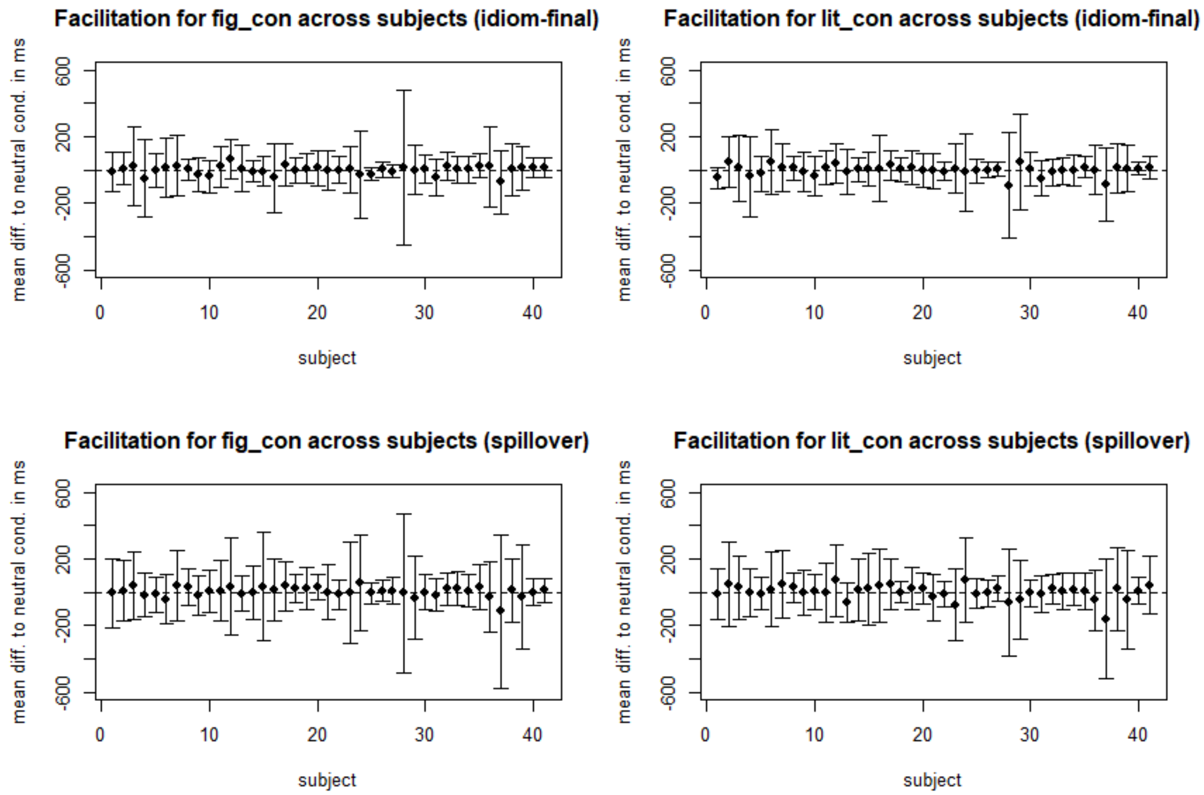

Figure C1: The mean differences between contexts per participant for both the idiom-final nouns (upper panels) and the spill-over words (lower panels). Error bars represent SDs.

The results of the linear mixed effects regression analysis on the idiom-final noun are presented in Table C2. This analysis revealed significant main effects of Non-verbal IQ and Word reading. The effect of non-verbal IQ was positive, indicating that participants with a higher non-verbal IQ were slower in reading the idiom-final noun than participants with a lower non-verbal IQ. In addition, we observed a facilitatory effect of word reading: Participants with better word reading skills were faster in reading the idiom-final noun than participants with poorer word reading skills. The effects of these participant-related predictors were not modulated by context.

Table C2: Idiom-final noun regression model with logged RTs as dependent variable (the no-context condition as the reference category).

**Fixed effects**	**β (SE)**	***t***	***p***	
Intercept	2.4455 (0.0324)	75.486	<0.001	***
Fig. biasing context (FBC)	-0.0013 (0.0048)	-0.274	0.784	
Lit. biasing context (LBC)	-0.0008 (0.0048)	-0.168	0.866	
Linguistic knowledge	-0.038 (0.0234)	-1.627	0.113	
FBC × Ling. knowledge	0.0059 (0.0059)	1.000	0.317	
LBC × Ling. knowledge	0.0088 (0.0059)	1.508	0.132	
Visual working memory (WM)	-0.0157 (0.0225)	-0.697	0.491	
FBC × Visual WM	-0.001 (0.0056)	-0.172	0.863	
LBC × Visual WM	0.0091 (0.0056)	1.621	0.105	
Processing speed	0.0184 (0.0216)	0.853	0.4	
FBC × Processing speed	0.0035 (0.0054)	0.64	0.522	
LBC × Processing speed	-0.0027 (0.0054)	-0.49	0.624	
Non-verbal IQ	0.0587 (0.0273)	2.148	0.039	*
FBC × Non-verbal IQ	-0.0081 (0.0068)	-1.189	0.235	
LBC × Non-verbal IQ	-0.0105 (0.0069)	-1.527	0.127	
Word reading	-0.0607 (0.0206)	-2.947	0.006	***
FBC × Word reading	0.0031 (0.0052)	0.596	0.551	
LBC × Word reading	0.005 (0.0052)	0.964	0.335	
Sentence compr. & pred. (SPC)	-0.0118 (0.0227)	-0.519	0.607	
FBC × SPC	0.0013 (0.0057)	0.233	0.816	
LBC × SPC	-0.0001 (0.0057)	-0.014	0.989	
Idiom transparency	-0.0014 (0.0049)	-0.289	0.775	
Idiom final noun frequency	0.0028 (0.0057)	0.491	0.629	
Idiom final noun length	-0.0017 (0.0044)	-0.384	0.705	
**Random effects**	**Variance**		**SD**	
Participant	0.0144		0.120	
Item	0.0004		0.020	
Residual	0.0114		0.107	

The results of the linear mixed effects regression analysis of the spill-over word are presented in Table C3. Similar to the idiom-final noun analysis, a significant main effect of word reading was observed, indicating that participants with better word reading skills were faster at reading the spill-over word than participants with poorer word reading skills. This effect was not modulated by context. Furthermore, the analysis revealed a significant interaction effect between linguistic knowledge and context (visualised in Figure C2). The effect of linguistic knowledge was significantly stronger for spill-over words in the no-context condition, than in the literally biasing context condition. In comparison to the figuratively biasing context condition, the effect of linguistic knowledge in the no-context condition was only marginally significantly stronger.

In sum, we observed no main effect of context. However, as predicted, we observed that individuals with better word reading abilities read idiom-final and spill-over words (in all three context conditions) faster than individuals with poorer word-reading skills. Non-verbal intelligence had a negative effect: In all three context conditions, individuals with higher scores read idiom-final words more slowly than individual with lower scores. Finally, we observed an interaction between Context and Linguistic knowledge such that spill-over words were read faster in the no-context condition (compared to the literally biasing condition) by individuals with larger rather than smaller linguistic knowledge.

Table C3: Spill-over word regression model with logged RTs as the dependent variable (the no-context condition as the reference category).

Fixed effects	β (SE)	t	p	
Intercept	2.519 (0.101)	24.943	<0.001	***
Fig. biasing context (FBC)	0.0039 (0.0058)	0.669	0.504	
Lit. biasing context (LBC)	0.0033 (0.0058)	0.574	0.566	
Linguistic knowledge	-0.0341 (0.0251)	-1.361	0.183	
FBC × Ling. knowledge	0.0131 (0.0071)	1.849	0.065	.
LBC × Ling. knowledge	0.0165 (0.0071)	2.327	0.02	*
Visual working memory (WM)	-0.0258 (0.0241)	-1.069	0.293	
FBC × Visual WM	0.0021 (0.0068)	0.314	0.753	
LBC × Visual WM	0.0045 (0.0068)	0.662	0.508	
Processing speed	0.0236 (0.0232)	1.018	0.316	
FBC × Processing speed	-0.0042 (0.0066)	-0.64	0.522	
LBC × Processing speed	-0.0041 (0.0066)	-0.632	0.527	
Non-verbal IQ	0.0569 (0.0293)	1.942	0.061	.
FBC × Non-verbal IQ	-0.0038 (0.0083)	-0.453	0.651	
LBC × Non-verbal IQ	-0.0006 (0.0083)	-0.077	0.939	
Word reading	-0.0599 (0.0221)	-2.707	0.011	*
FBC × Word reading	0.0025 (0.0063)	0.394	0.694	
LBC × Word reading	0.0011 (0.0063)	0.178	0.859	
Sentence compr. & pred. (SPC)	-0.0197 (0.0244)	-0.809	0.424	
FBC × SPC	-0.0055 (0.0069)	-0.793	0.428	
LBC × SPC	-0.0034 (0.0069)	-0.496	0.62	
Idiom transparency	0.001 (0.0078)	0.132	0.897	
Spill-over word frequency	-0.0064 (0.0174)	-0.368	0.716	
Spill-over word length	0.0024 (0.005)	0.481	0.635	
**Random effects**	**Variance**		**SD**	
Participant	0.0165		0.129	
Item	0.0013		0.036	
Residual	0.0167		0.129	

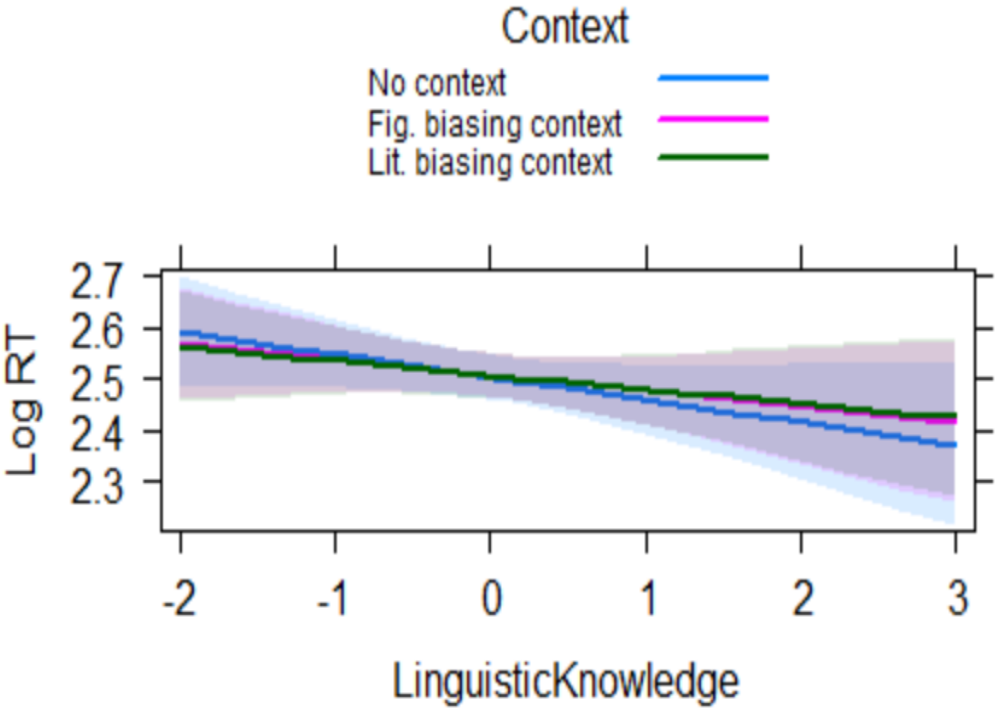

Figure C2: The interaction between Context and Linguistic knowledge for the spill-over word. The error bands represent the 95% confidence interval.
